# Fibroma with minor sex cord elements: a case report and review of the literature

**DOI:** 10.1186/1757-1626-1-264

**Published:** 2008-10-23

**Authors:** Vibha Kawatra, Parul Gupta, Nita Khurana

**Affiliations:** 1 Department of Pathology, Maulana Azad Medical College and Lok Nayak Hospital, Bahadur Shah Zafar Marg, New Delhi 110002, India

## Abstract

**Introduction:**

Ovarian fibroma is a rare neoplasm and the one with focal proliferations of sex cord type elements is rarer. Histopathological importance lies in the recognition of this entity due to the diagnostic dilemmas that these tumors can pose. To the best of our knowledge only 9 cases of ovarian fibroma with minor sex cord elements have been documented in world literature till date.

**Case presentation:**

We report the case of a 65-year-old Indian female who presented with postmenopausal bleeding and pain abdomen. An ultrasound scan of the abdomen revealed a right ovarian mass. Histologically, the ovarian tumor was a fibroma with minor sex cord elements.

**Conclusion:**

Clinical manifestations of a hyperestrogenic state in postmenopausal female should raise suspicion of this entity in the mind of a physician. Also a thorough evaluation of an ovarian fibroma would help detection of minor sex cord elements within the tumor and thus be a stepping stone for better evaluation of the pathogenesis and clinical behaviour of these tumors. A close follow up of the patient should be done as hyperestrogenemia may predispose to endometrial cancer.

## Introduction

Ovarian fibroma with minor sex cord elements is a rare entity that was first described by Young and Scully in 1983. It is defined as a predominantly fibromatous or a thecomatous tumor containing scattered minor sex cord elements in less than 10% of the tumor area.[1] Only 9 cases of ovarian fibroma with minor sex cord elements have been reported till date. [1-4]

We report case of a 65 year old female with complaints of bleeding and pain abdomen for the past 6 months. The uterus was enlarged and abdominal ultrasonography revealed a right ovarian solid mass. Simple endometrial hyperplasia with endometrial polyp was noted and this probably was a result of hyperestrogenic state due to hormone production by the ovarian tumor. This could predispose to endometrial cancers as well. Though these tumors like, fibromas are thought to behave in a benign fashion, concrete evidence in their clinical behaviour is still a challenge that needs further evaluation.

The importance of recognition of ovarian fibroma with minor sex cord elements lies in the diagnostic dilemmas that they can pose, as they have on various occasions been mistaken to be Brenner tumors, adenofibromas, or metastatic carcinoids.

We herein review the clinicopathological features of this rare entity.

## Case presentation

A 65 year old Indian female, gravida 7, para 7, non smoker, non alcoholic, housewife weighing 60 kgs, height 5'6" presented to the gynecology outpatient with complaints of bleeding and pain abdomen for the past 6 months. No history of any drug intake or previous medical problems or family history of any disease was present. On vaginal examination uterus was enlarged to 8 weeks in size, retroverted with clear bilateral fornices. Abdominal ultrasonography revealed a right ovarian solid mass. The left ovary was normal. Endometrial curettings revealed simple glandular hyperplasia without cytological atypia. A clinical diagnosis of dysfunctional uterine bleeding was made. A total abdominal hysterectomy with bilateral salpingo-oophorectomy was performed.

**Figure 1:**
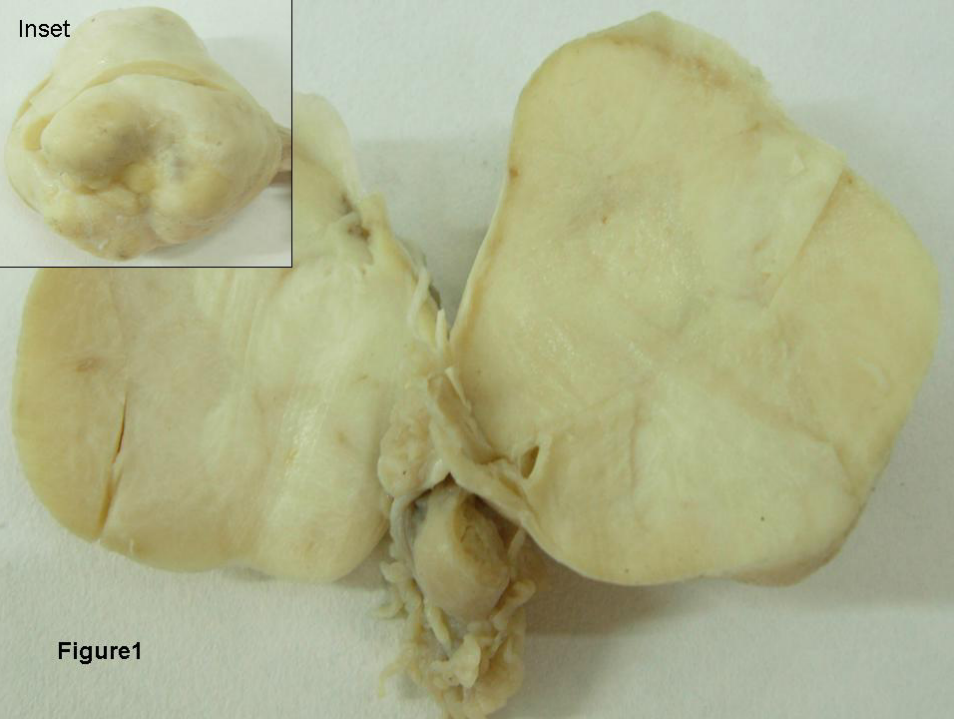
**Cut surface shows a firm grey white solid mass with focal yellowish areas with compressed normal ovarian parenchyma at the periphery.** {Inset} Encapsulated right ovary measuring 5 × 4 × 2 cm.

Grossly we received a specimen of uterus with cervix along with bilateral adnexa. The right ovary measured 5 × 4 × 2 cm. It was globular, nodular and externally encapsulated with no capsular breech (Fig 1a). Sectioning of the ovary revealed a predominantly firm grey white solid mass with focal yellowish firm 0.2 × 0.2 cm areas with compressed normal ovarian parenchyma at the periphery (Fig 1b). Bilateral fallopian tubes and the left ovary were grossly unremarkable. Sectioning of uterus revealed an endometrial polyp measuring 2 × 0.5 × 0.5 cm. The myometrium on serial slicing showed a focus of hemorrhage and an intramural fibroid.

**Figure 2:**
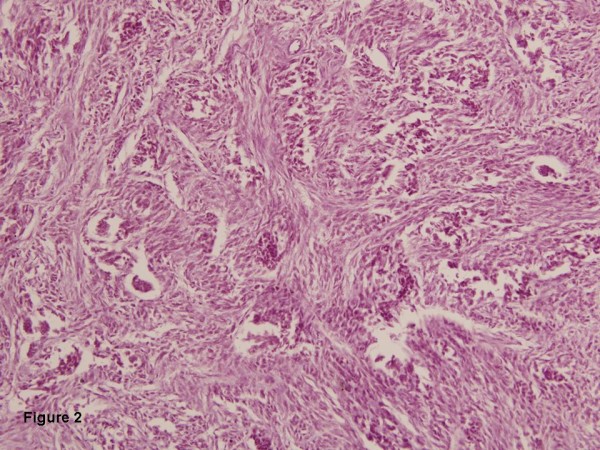
Highly cellular tumor composed of uniform spindle cells arranged in sheets and intersecting fascicles with scattered nests and cords of relatively uniform large cells with inconspicuous nucleoli.

Histologically, the right ovarian tumor was highly cellular composed of uniform spindle cells arranged in sheets and intersecting fascicles (Fig 2a). The cells had scant to moderate amount of eosinophilic cytoplasm with elongated nuclei with tapered ends and 1–2 inconspicuous nucleoli. Mitotic figures ranged from 0 to 1 per 10 high powerfields. Dispersed among these cells were few scattered clusters of relatively uniform large cells with inconspicuous nucleoli, arranged in poorly defined nests and cords (Fig 2b). These small aggregates were sharply demarcated from the surrounding stroma and formed less than 5% of the tumor area.

The spindled out cells stained blue with Masson Trichrome stain. Immunohistochemically, the spindled cells were diffusely positive for smooth muscle actin (SMA) and weakly expressed vimentin and inhibin. These spindled cells were negative for epithelial membrane antigen (EMA) and cytokeratin. The uniform large cells in nests and cords were strongly immunoreactive for inhibin and negative for vimentin, SMA and EMA. Based on these above findings a final diagnosis of fibroma with minor sex cord elements was made.

## Discussion

Ovarian stromal tumor with minor sex cord elements is a rare neoplasm, which was first described by Young and Scully as a predominantly fibromatous or a thecomatous tumor containing scattered minor sex cord elements in less than 10% of the tumor area.[1] The minor sex cord elements are seen as small nests or tubules of cells resembling granulosa cells, Sertoli cells, or indifferent cells of sex cord type.

To the best of our knowledge only 9 cases of ovarian fibroma with minor sex cord elements have been documented in world literature till date. [1-7]

Sex cord-stromal tumors represent a heterogeneous group of lesions, including Leydig cell tumor, Sertoli cell tumor, mixed Sertoli-Leydig cell tumor, granulosa cell tumor, and a poorly defined tumor group referred to as unclassified sex cord-stromal tumors.[8]

The stromal component is usually fibromatous or thecomatous. They are firm, solid, grey white to grey yellow in colour. Microscopically they are composed of spindled cells, arranged in intersecting fascicles with variable amount of collagen deposition. The nucleus of the stromal cells is elongated with tapering ends and no prominent nucleoli. The term "minor" component of sex cord elements is defined as sex cord elements occupying no more than 10% of the area of the tumor on any slide.[1] The individual aggregate of these minor sex cord elements should not be greater than 0.45 mm. Immunihistochemically, the minor sex cord elements are positive for inhibin, calretinin, CD99, CD56, antikeratin antibody KL1 and MIC. [2,6]

Their importance lies in the diagnostic dilemmas that they can pose, as they have on various occasions been mistaken to be Brenner tumors, adenofibromas, or metastatic carcinoids. Sex cord elements can be distinguished by their strong immunoreactivity to inhibin. The epithelial aggregates of Brenner tumor are composed of transitional cells, mucinous cells or both and sometimes have a central lumen containing eosinophilic secretions. The glands of adenofibroma are abundant, larger, and tubular and more variable in size than the uniform tubules of minor sex cord elements.[1]

In the study by Young and Scully[1] (1983), 2 of the 7 cases of fibromas with minor sex cord elements showed evidence of hormone overproduction with the presence of well-differentiated adenocarcinoma in the endometrium. Also, in the study by Zhang et al[2], 23 of the fibrothecomatous tumors showed the coexistence of cystic hyperplasia, atypical hyperplasia, or adenocarcinoma of the endometrium. These findings were attributed to production of estrogenic hormone by the tumors. Similarly in our case, the findings of simple hyperplasia with endometrial polyp formation in a postmenopausal woman may be the result of a hyperestrogenic state due to hormone production by the ovarian tumor.

These tumors like, fibromas are thought to behave in a benign fashion and have a good prognosis. Our patient in one year post surgery follow up has been asymptomatic and healthy.

Fibroma with minor sex cord element is a rare pathological entity among the unclassified sex cord stromal tumors and awareness of its occurrence is important for a practicing pathologist. Detection of these tumors would serve a basis for further research in their pathogenesis and provide a better insight of the clinical behaviour of these rare neoplasms.

## Conclusion

Clinical manifestations of a hyperestrogenic state in postmenopausal female should raise suspicion of this rare entity in the mind of a physician. Also for a pathologist, a thorough evaluation of an ovarian fibroma would help detection of minor sex cord elements within the tumor and thus be a stepping stone for better evaluation of the pathogenesis and clinical behaviour of these tumors. A close follow up of the patient should be done as hyperestrogenemia may predispose to endometrial cancer.

This case has been presented to enlighten the physicians and pathologists of the rare gynecological neoplasms and pave way to further research in this field.

## Competing interests

The authors declare that they have no competing interests.

## Authors' contributions

VK analyzed and interpreted the patient data regarding the disease. VK, PG and NK performed the histological examination of the ovary, and all contributed in writing the manuscript. All authors read and approved the final manuscript.

## Consent

Written informed consent was obtained from the patient for publication of this case report and accompanying images. A copy of the written consent is available for review by the Editor-in-Chief of this journal.
